# Precursor B‐cell lymphoblastic lymphoma presenting as concurrent enlarging masses on the scalp and postauricular region in a 13‐year‐old boy

**DOI:** 10.1002/kjm2.12811

**Published:** 2024-02-16

**Authors:** Wei‐Yao Wang, Sheau‐Fang Yang, Yu‐Wen Cheng, Yang‐Yi Chen

**Affiliations:** ^1^ Department of Dermatology Kaohsiung Medical University Hospital Kaohsiung Taiwan; ^2^ Department of Dermatology College of Medicine, Kaohsiung Medical University Kaohsiung Taiwan; ^3^ Department of Pathology Kaohsiung Medical University Hospital Kaohsiung Taiwan; ^4^ Department of Neurosurgery Kaohsiung Veterans General Hospital Kaohsiung Taiwan; ^5^ Graduate Institute of Clinical Medicine, College of Medicine Kaohsiung Medical University Kaohsiung Taiwan

A 13‐year‐old Taiwanese boy presented with a 6‐month history of enlarging, asymptomatic scalp and postauricular masses. No fever, weight loss, or night sweats were present. Physical examination showed a dome‐shaped, firm, non‐tender, erythematous mass measuring 5 cm in diameter on the right parietal scalp (Figure 1A,B), and enlarged lymph nodes measuring 1.5 cm in the right postauricular region (Figure 1C). Ultrasonography showed abnormal lymph nodes with peripheral vascularity in the hypodermis (Figure 1D). Laboratory investigations, including lactate dehydrogenase, yielded unremarkable results. Serologic tests for Epstein–Barr virus and human immunodeficiency virus were negative.

**FIGURE 1:**
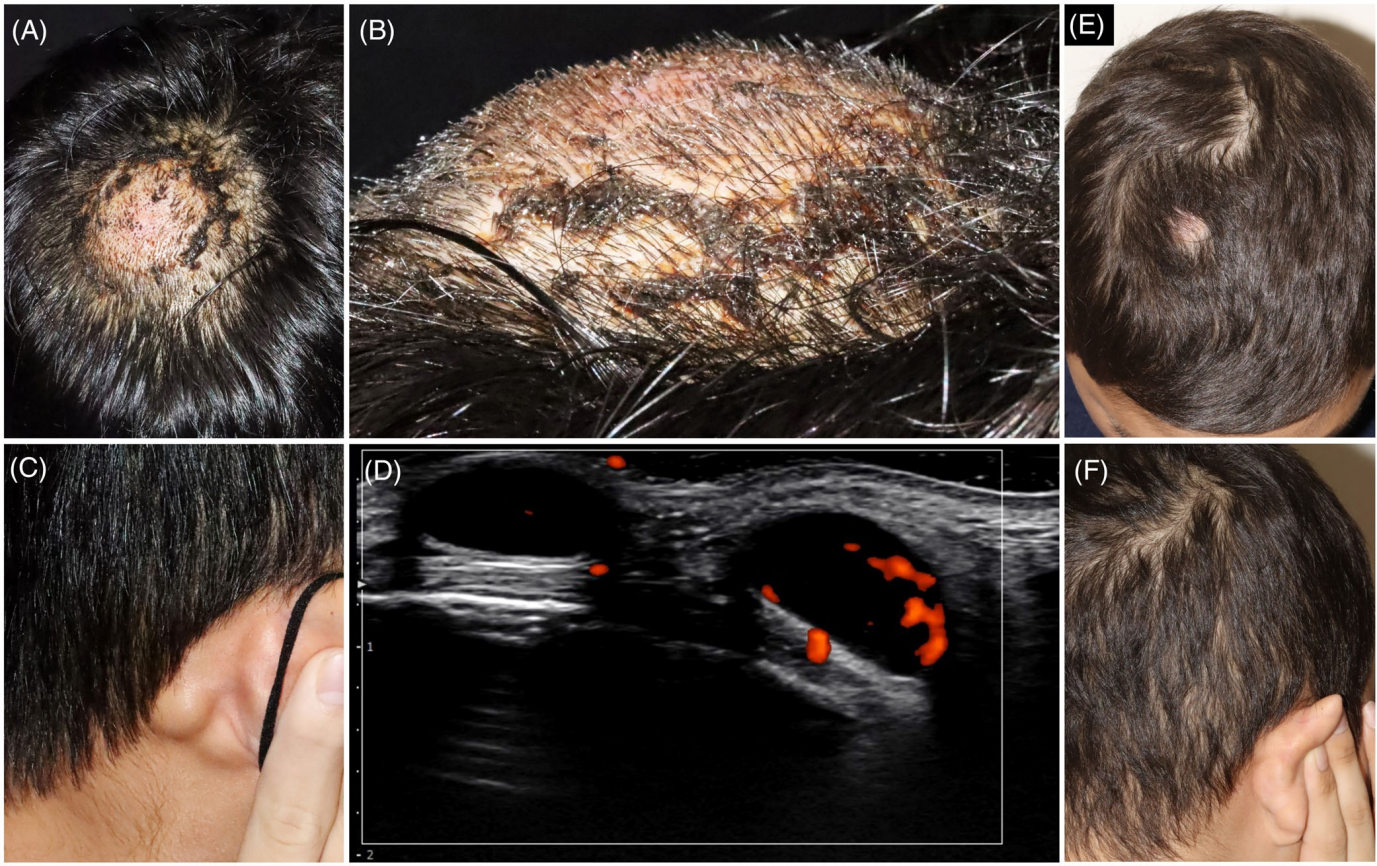
Clinical and ultrasonographic images. (A) A dome‐shaped, firm, non‐tender, erythematous mass, measuring 5 cm in diameter, was located on the right parietal scalp. Mud‐like residues of Chinese herbal ointment were noted on the scalp. (B) Close‐up lateral view of the scalp mass. (C) Enlarged lymph nodes, measuring 1.5 cm, were detected in the right postauricular region. (D) Ultrasonography of lymph nodes revealed two oval anechoic structures with peripheral vascularity in the hypodermis. (E) The scalp mass had completely resolved at the 24‐week follow‐up, leaving only an alopecic patch. (F) The right postauricular lymph nodes exhibited complete resolution at the 24‐week follow‐up.

The scalp biopsy revealed a dense dermal infiltrate extending into the subcutaneous tissue, along with a grenz zone. This infiltrate consisted of uniform, small‐round‐blue cells with evident mitotic figures (Figure S1A,B). The lymph node biopsy disclosed obliteration of architectural structures by small‐round‐blue cells. (Figure S1C). The neoplastic cells exhibited positive immunohistochemical staining for terminal deoxynucleotidyl transferase (TdT), CD79a, CD10, and CD20 (Figure S1D,E).

Brain magnetic resonance imaging, abdominal ultrasonography, whole‐body bone scan, and serum immunofixation electrophoresis revealed normal results. Whole‐body positron emission tomography scan revealed elevated fluorodeoxyglucose uptake in the right parietal scalp mass and postauricular lymph nodes. The bone marrow aspiration showed hypocellularity but preserved trilineage hematopoietic cells, with blasts constituting ~5% of the cell population. A diagnosis of precursor B‐cell lymphoblastic lymphoma (pB‐LBL) with cutaneous and nodal involvement was established, staging the disease as stage II following the St. Jude/Murphy staging system.

The patient initiated a 9‐week course of multiagent induction chemotherapy following the Taiwan Pediatric Oncology Group protocol for pB‐LBL. This protocol included prednisolone, vincristine, epirubicin, asparaginase, cyclophosphamide, cytarabine, mercaptopurine, and methotrexate. After the initial induction chemotherapy, a 7‐week consolidation therapy with mercaptopurine, methotrexate, and cytarabine was administered, followed by reinduction chemotherapy. This comprehensive treatment results in complete resolution at the 24‐week follow‐up (Figure 1E,F).

PB‐LBL is a rare subtype of non‐Hodgkin lymphoma (NHL) that arises from immature B lymphocytes, constituting only 1% of all hematological malignancies in children.^1^, ^2^, ^3^, ^4^ It primarily affects children and adolescents, with a slight male preponderance.^2^, ^3^, ^4^ PB‐LBL often manifests in localized extramedullary sites, including lymph nodes, bones, skin, and mediastinum.^1^, ^2^, ^3^ In pB‐LBL, the occurrence of skin or subcutaneous involvement in the head and neck region, along with lymph node participation, is uncommon, observed in ~4.7% of cases.^1^ It typically presents as multiple red‐to‐purple nodules/masses. Various pediatric scalp disorders manifest as gradually enlarging masses and should be considered as differential diagnoses. These include other NHL, hemangioma, lymphangioma, neurofibroma, Langerhans cell histiocytosis, desmoid tumors, cranial fasciitis, and kimura disease.^3^, ^5^ Histopathological examination and immunohistochemical studies are necessary for an accurate diagnosis. Immunohistochemically, the lymphoblasts typically express markers such as TdT, cytoplasmic CD79a, and CD19, indicating an early B‐lymphoid lineage.^2^, ^3^, ^4^

This case highlights the significance of considering pB‐LBL as a potential diagnosis when encountering progressively enlarging scalp masses in children. Early identification is critical, as prompt initiation of multi‐agent chemotherapy often leads to a favorable prognosis, with reported 5‐year overall survival rates reaching up to 90%.^1^, ^4^

## CONFLICT OF INTEREST STATEMENT

All authors declare no conflict of interest.

## Supporting information


**Supplementary Figure S1.** Histopathological and immunohistochemical images. (A) The scalp biopsy showed a dense dermal infiltrate that dissected through the collagen bundles and extended into the subcutaneous tissue, along with a grenz zone (hematoxylin and eosin, ×10). (B) The dense dermal infiltrate consists of uniform, small‐round‐blue cells with hyperchromatic nuclei and scant cytoplasm (hematoxylin and eosin, ×200). (C) The lymph node biopsy revealed the obliteration of architectural structures by small‐round‐blue cells. (hematoxylin and eosin, ×200). (D) The neoplastic cells displayed diffuse positive nuclear staining for TdT (TdT immunostaining, ×200). (E) These neoplastic cells displayed diffuse positive cytoplasmic staining for CD79a (CD79a immunostaining, ×200).
