# Supplementary figures and images for: Precursor B‐cell lymphoblastic lymphoma presenting as concurrent enlarging masses on the scalp and postauricular region in a 13‐year‐old boy
Source: Kaohsiung J Med Sci. 2024 Feb 16;40(5):509–10. doi: 10.1002/kjm2.12811 (PMC11895603; doi:10.1002/kjm2.12811)

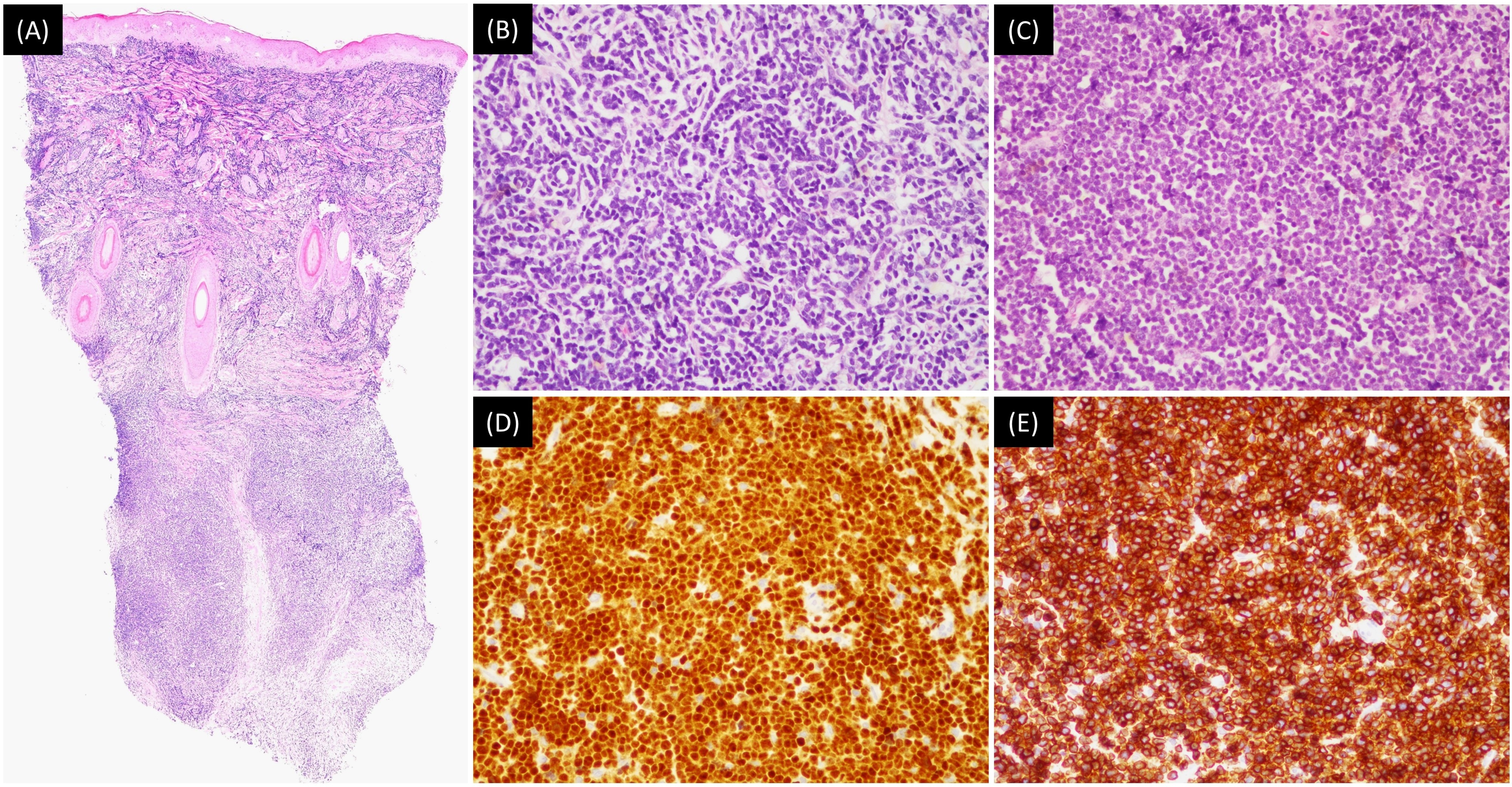

Supplement: Supplementary file 1 — Supplementary Figure S1. Histopathological and immunohistochemical images. (A) The scalp biopsy showed a dense dermal infiltrate that dissected through the collagen bundles and extended into the subcutaneous tissue, along with a grenz zone (hematoxylin and eosin, ×10). (B) The dense dermal infiltrate consists of uniform, small‐round‐blue cells with hyperchromatic nuclei and scant cytoplasm (hematoxylin and eosin, ×200). (C) The lymph node biopsy revealed the obliteration of architectural structures by small‐round‐blue cells. (hematoxylin and eosin, ×200). (D) The neoplastic cells displayed diffuse positive nuclear staining for TdT (TdT immunostaining, ×200). (E) These neoplastic cells displayed diffuse positive cytoplasmic staining for CD79a (CD79a immunostaining, ×200). [file KJM2-40-509-s001.jpeg]
